# In Silico Investigation of the Molecular Mechanism of PARP1 Inhibition for the Treatment of BRCA-Deficient Cancers

**DOI:** 10.3390/molecules28041829

**Published:** 2023-02-15

**Authors:** Fengqin Yan, Zhenfu Fu, Guo Li, Zhiguo Wang

**Affiliations:** 1 Department of Radiotherapy, The Cancer Hospital of the University of Chinese Academy of Sciences (Zhejiang Cancer Hospital), Institute of Basic Medicine and Cancer (IBMC), Chinese Academy of Sciences, Hangzhou 310022, China; 2 Department of Biochemistry and Molecular Biology, Hainan Medical University, Haikou 571199, China; 3 Hainan Province Clinical Medical Center, Hainan Hospital Affiliated to Hainan Medical University, Haikou 571199, China; 4 Institute of Ageing Research, School of Basic Medical Sciences, Hangzhou Normal University, Hangzhou 311121, China

**Keywords:** PARP1 inhibition, BRCA deficiency, *PARP1* G-quadruplex, molecular mechanism, molecular dynamics

## Abstract

The protein PARP1, which plays a crucial role in DNA repair processes, is an attractive target for cancer therapy, especially for BRCA-deficient cancers. To overcome the acquired drug resistance of PARP1, *PARP1* G-quadruplex (G4) identified in the PARP1-promotor region is gaining increasing attention. Aiming to explore the molecular mechanism of PARP1 inhibition with *PARP1* G4 and PARP1 as potential targets, a comparative investigation of the binding characteristics of the newly identified G4 stabilizer MTR-106, which showed modest activity against talazoparib-resistant xenograft models and the FDA-approved PARP1 inhibitor (PARPi) talazoparib, were performed through molecular simulations. Combined analyses revealed that, relative to the groove binding of talazoparib, MTR-106 induced the formation of a sandwich framework through stacking with dT_1_ and the capping G-pair (dG_2_ and dG_14_) of *PARP1* G4 to present largely enhanced binding affinity. For the binding with PARP1, although both were located in the catalytic pocket of PARP1, MTR-106 formed more extensive interactions with the surrounding PARP1 residues compared to talazoparib, in line with its increased binding strength. Importantly, vdW interaction was recognized as a decisive factor in the bindings with *PARP1* G4 and PARP1. Collectively, these findings demonstrated the ascendancy of MTR-106 over talazoparib at the atomic level and revealed that the dual targeting of *PARP1* G4 and PARP1 might be pivotal for PARPi that is capable of overcoming acquired drug resistance, providing valuable information for the design and development of novel drugs.

## 1. Introduction

Poly(ADP-ribose) polymerases (PARPs) catalyze the transfer of single or multiple ADP-ribose units from nicotinamide adenine dinucleotide (NAD^+^) to target proteins [1], resulting in the modulation of their enzyme activity and cellular localization and the formation of multimeric protein complexes [2,3]; these processes are named MARylation or PARylation, respectively [4]. According to the domain arrangement, including the DNA-dependent tankyrases, CCCH zinc finger, and macro-domain, the human PARP family comprises 17 members, belonging to four subfamilies [5]. Poly(ADP-ribose) polymerase-1 (PARP1), as an abundant chromatin-associated nuclear protein, is vital for genomic integrity [1,2]. The PARP1 gene activates in a DNA-dependent way and acts as one of the earliest cellular responses to DNA damage [6]. It is involved in the repair of single-stranded DNA breaks, and its inhibition leads to synthetic lethality [7,8], making PARP1 an attractive target for tumor cells with defective homologous recombination (HR) pathways [9].

Through HR, breast-cancer-susceptibility proteins 1 and 2 (BRCA1 and BRCA2) are responsible for the repair of the double-stranded DNA breaks caused by the PARP1-inhibition-induced accumulation of DNA lesions [10], and the inhibition of PARP1 sensitizes BRCA1/2-deficient cancer cells to cell-cycle arrest and apoptosis [11,12]. Therefore, PARP1 is one of the most intensively investigated targets for treating cancers associated with BRCA1/2 deficiency [13,14]. The US Food and Drug Administration (FDA) and European Medicines Agency (EMA) have approved PARP1 inhibitors (PARPi), including olaparib (2014), rucaparib (2016), and niraparib (2017) for the treatment of BRCA-mutated ovarian-cancer patients [9]. In addition, talazoparib was approved in 2018 by the US FDA for the treatment of breast cancer with germline BRCA mutations [15]. Despite the promising clinical benefits observed with PARPi monotherapy, acquired resistance to PARPi can develop via three general mechanisms: drug-target-related effects, such as the upregulation of drug efflux pumps or mutations in PARP or functionally related proteins; the restoration of HR owing to the restoration of BRCA1/2 function; or the loss of DNA end-protection and/or the restoration of replication-fork stability, which significantly limits their applications [16,17,18,19]. Therefore, alternative strategies, such as PARP1-expression regulation alone or in combination with PARPi, are urgently needed.

Studies using G-quadruplex-specific sequencing and nuclear magnetic resonance (NMR) have determined a G-quadruplex (G4) structure in the promoter region of the *PARP1* gene [20]. The intramolecular *PARP1* G4 has a three-layered bulge-containing (3 + 1) hybrid scaffold, providing opportunities for selective targeting by chemical stabilizers. Stabilizing G4 structures formed in oncogene promotors, such as *c-MYC*, *c-KIT*, and *BCL-2*, show a promising effect on the inhibition of oncogene expressions, leading to cell-cycle arrest and the apoptosis of cancer cells [21,22,23,24]. Therefore, the stabilization of *PARP1* G4 could be a promising strategy for treating BRCA-deficient cancers. Furthermore, G4 stabilizers have been reported to kill HR-deficient cancer cells selectively. The DNA G4 stabilizer, CX-5461, shows a different sensitivity spectrum to PARPi and demonstrates therapeutic efficacy in PARPi-resistant high-grade serous ovarian cancer [25]. In addition, PARP1 inhibitors, including LOM1392 and MK4827, were observed binding and stabilizing the *PARP1* G4 [26]. These findings indicated that dual-targeting compounds for G4 and PARP1 may represent a new solution for drug development targeting BRCA-deficient cancers.

Recently, a novel quinolone-amide compound named MTR-106 was developed. It acted as a G4 stabilizer and displayed advanced antiproliferative activity, increased DNA damage, and increased cell-cycle arrest and the apoptosis of BRCA-deficient cancer cells. Importantly, MTR-106 shows activity against talazoparib-resistant xenograft mouse models [27]. However, the molecular mechanism underlying the remarkable bioactivities of MTR-106 remains to be determined. In the current study, by employing combined molecular docking and molecular dynamics (MD) simulations, a comparative study on the binding mechanisms of MTR-106 and talazoparib with *PARP1* G4 and PARP1 was performed. Through comprehensive analyses, including principal components analysis (PCA), the G4 stabilization effect, hydrogen bonding, non-covalent interactions (NCI), molecular mechanics/generalized Born surface area (MM/GBSA) calculation, and residue-based free energy decomposition, the binding features of MTR-106 and talazoparib with *PARP1* G4 and PARP1 were characterized. The pharmacokinetic and toxicity profiles (absorption, distribution, metabolism, excretion, and toxicity—ADMET) of both compounds were evaluated. The results support experimental findings from assays of cellular and animal models and provide valuable information for designing novel G4 stabilizers and PARPi with desired biological properties for the treatment of BRCA-deficient cancers.

## 2. Computational Procedures

### 2.1. Data

**Figure 1:**
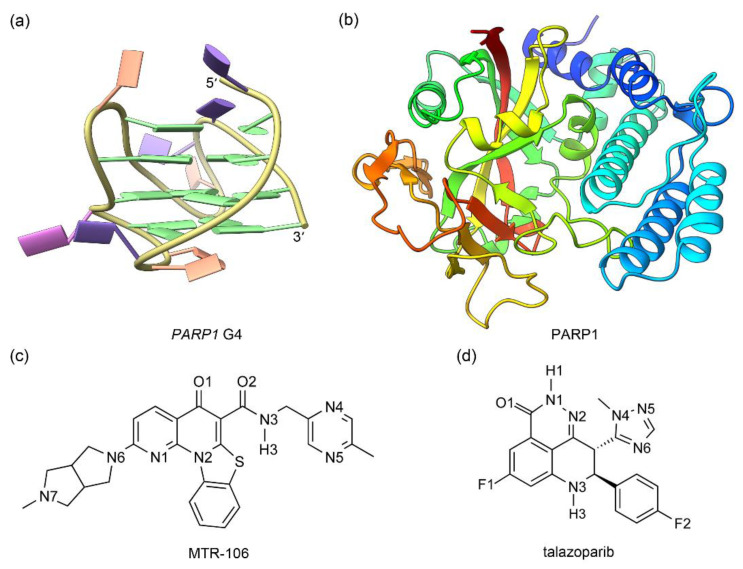
The structures of *PARP1* G4 (**a**) and PARP1 catalytic domain (**b**) together with the ligands of MTR-106 (**c**) and talazoparib (**d**). In *PARP1* G4, the nucleotide bases of adenine (A), cytosine (C), guanine (G), and thymine (T) are colored pink, orange, green, and purple, respectively (**a**). For the catalytic domain of PARP1, blue fades to red as the structure moves from the N-terminal to the C-terminal (**b**).

The solution NMR structure of human (3 + 1) hybrid *PARP1* G4 and the crystal structure of the catalytic domain of human PARP1 in complex with talazoparib were retrieved from PDB data bank with the IDs of 6AC7 [20] and 7KK3 [28], respectively (Figure 1a,b). Central potassium ions are vital to the structural stability of G4s [29,30,31]; therefore, in addition to the NMR *PARP1* G4, a model with two K^+^ intercalated between adjacent G-tetrads (denoted as G4(K^+^)) was generated by using the UCSF ChimeraX software (version 1.5) [32]. The G4 and G4(K^+^) were subjected to MD simulations to obtain their equilibrated conformations. For the PARP1 structure, the undetermined residues located in the peripheral regions were built using the loop-modeling function of UCSF ChimeraX. The structures of MTR-106 and talazoparib were built with GaussView software and were optimized at the level of DFT B3LYP/6-31G(d) [33,34] (Figure 1c,d). For both compounds, the atomic charges were calculated using the restricted electrostatic potential (RESP) method with Gaussian 03 at the level of HF/6-31G(d) [34], and the other force-field parameters were generated from the generalized amber force field (GAFF) with the antechamber module [35].

### 2.2. Molecular Docking

Molecular-docking calculations were performed using the AutoDock Vina 1.2.0 software [36]. The receptors of *PARP1* G4 and G4(K^+^) and the ligands of MTR-106 and talazoparib were prepared with AutoDockTools-1.5.6 software [37]. The non-polar hydrogen atoms were merged, and Gasteiger charges were added for the receptors and ligands. In the docking calculations, the rotatable bonds of MTR-106 and talazoparib were flexible, while *PARP1* G4 and G4(K^+^) were set as rigid receptors. Since we had no prior knowledge of the binding domain of the newly determined *PARP1* G4, to avoid missing any possible binding forms of MTR-106 and talazoparib, the grid box that defines the binding regions of ligands was set to be large enough to encompass the whole structure of *PARP1* G4/G4(K^+^) in the docking calculations, i.e., a cubic box centered at the G-quadruplex geometric center comprising 30 × 30 × 30 grids with a grid spacing of 1.0 Å was used. Other parameters were set as defaults. Five rounds of docking calculations were performed for each ligand in order to obtain the energetically favored and highly probable binding conformations [38]. In the docking calculations of PARP1 and MTR-106, the bound talazoparib and the crystal waters were firstly deleted from the structure of PARP1. Polar hydrogen atoms and the atomic Gasteiger charges were added, and the resultant PARP1 served as the receptor. The binding region was defined by using the location of talazoparib as a reference.

### 2.3. Molecular Dynamics

Amber 20 software was applied in the MD simulations of all models [39,40]. Each of the apo G4/G4(K^+^) and the binding complexes of *PARP1* G4/G4(K^+^)–MTR106/talazoparib was placed at the center of a truncated octahedron box of TIP3P water molecules at a margin distance of 10.0 Å. To keep the modeling systems in electrical neutrality, environmental potassium and/or chloride ions were added to apo G4/G4(K^+^), G4/G4(K^+^)–MTR106/talazoparib, and PARP1–MTR106/talazoparib, respectively. The previously validated FF99SB force fields with parmbsc1 and χ_OL3+OL15_ modifications were applied for *PARP1* G4/G4(K^+^) [41,42]. The calibrated parameter (radius 1.705 Å, well depth 0.1936829 kJ·mol^−1^) and standard Amber parameter (radius 2.658 Å, well depth 0.00328 kJ·mol^−1^) were used for the central and environmental K^+^ ions, respectively [43]. For PARP1, the Amber FF99SB force field was applied [44]. Each model was firstly energy-minimized by 5000 steps of steepest descent minimization with a harmonic constraint of 500 kcal mol^−1^ Å^−2^ imposed on G4/G4(K^+^) and the binding complexes, followed by 5000 steps of conjugated gradient minimization without any constraints. Next, the system was gradually heated from 0 to 300 K under the NVT ensemble for 500 ps, with a weak constraint of 10 kcal mol^−1^ Å^−2^ imposed on G4/G4(K^+^) or the binding complexes. Each model was subsequently subjected to an equilibrium simulation for 1 ns by removing all constraints. Finally, a long-time-scale production simulation for each model was conducted under the NPT ensemble. Specifically, to ensure that all the systems reached MD equilibration, the apo *PARP1* G4/G4(K^+^), G4/G4(K^+^)–MTR106/talazoparib, and PARP1–MTR106/talazoparib were simulated for 1000 ns, 1000~1500 ns, and 300 ns, respectively. In all MD simulations, parameters were set by following our previous reports [43,45]. MD trajectories were recorded at an interval of 1 ps for the structural and energetic analyses.

### 2.4. Principal Components Analysis

A PCA was performed to describe the essential motions of *PARP1* G4/G4(K^+^) and PARP1 by removing the overall translational and rotational movements from MD trajectories [46]. Based on 5000 frames evenly extracted from the last 200 ns of MD trajectories, a PCA was carried out on the backbones of G4/G4(K^+^) and PARP1 for each model using the CPPTRAJ module of AmberTools. The graphical summary of motions along a specific eigenvector was shown in a porcupine plot generated by using the VMD software (version 1.9.4) [47].

### 2.5. Noncovalent Interactions

The NCIplot [48] calculations were carried out with a step size of 0.10 to visualize the interacting regions between G4/G4(K^+^)/PARP1 and the binding ligands. The reduced gradients were rendered as an isosurface in VMD, using an isovalue of 0.3 au.

### 2.6. Binding-Free-Energy Analysis

The binding free energies between G4/G4(K^+^)/PARP1 and the binding ligands were obtained from the MM/GBSA calculations. For each of the binding-complex trajectories, 500 snapshots were evenly extracted from the last 200 ns of MD trajectory for the calculation. The binding-free-energy value is equal to the free-energy difference between the binding complex (G_complex_) and the sum of receptor (G_rec_) and ligand (G_lig_) as follows:

∆G_bind_ = G_complex_ − (G_rec_ + G_lig_) (1)

Each of them can be calculated with the following equation:

∆G_bind_ = ∆H − T∆S ≈ ∆E_MM_ + ∆G_solv_ − T∆S (2)

where ∆E_MM_ is the molecular mechanical energy of the gas phase, ∆G_solv_ is the solvation free energy, and T∆S is the contribution of entropy. The ∆E_MM_ comprises contributions from electrostatic energy (∆E_ele_), van der Waals (vdW) interaction energy (∆E_vdW_), and internal-strain energy (∆E_int_) which includes bonds, angles, and dihedral energies that can be ignored in our systems:

∆E_MM_ = ∆E_ele_ + ∆E_vdW_ + ∆E_int_ (3)

The ∆G_solv_ contains contributions from a polar part (∆G_GB_) and a non-polar (∆G_SA_) part:

∆G_solv_ = ∆G_GB_ + ∆G_SA_ (4)

The ∆G_GB_ was estimated by the generalized Born (GB) model with the interior and exterior dielectric constants set to 4 and 80, respectively [49,50]. The nonpolar solvation terms were calculated according to the LCPO algorithm:

∆G_SA_ = γ∆SASA + β (5)

where γ and β were set to 0.0072 kcal·mol^−1^·Å^−2^ and 0, respectively [51]. Therefore, the binding free energy was calculated as follows:

∆G_bind_ = ∆E_ele_ + ∆E_vdW_ + ∆E_int_ + ∆G_GB_ + ∆G_SA_ − T∆S (6)

The entropic contribution (T∆S) was evaluated through normal mode analysis (NMA) [52]. Due to the expensive computational cost of NMA, 200 snapshots were evenly extracted from the last 200 ns of MD trajectory for the entropy calculations [43].

### 2.7. ADMET Analysis

The ADMET properties of MTR-106 and talazoparib were predicted using the online servers of admetSAR2 (http://lmmd.ecust.edu.cn/admetsar2 (accessed on 2 February 2023)) [53] and ProTox-II (https://tox-new.charite.de/protox_II (accessed on 2 February 2023)) [54]. The bioavailability radar of both compounds was generated with the SwissADME server (http://www.swissadme.ch (accessed on 2 February 2023)) [55].

## 3. Results

### 3.1. Conformational Characteristics of the Apo PARP1 G4/G4(K^+^)

**Figure 2:**
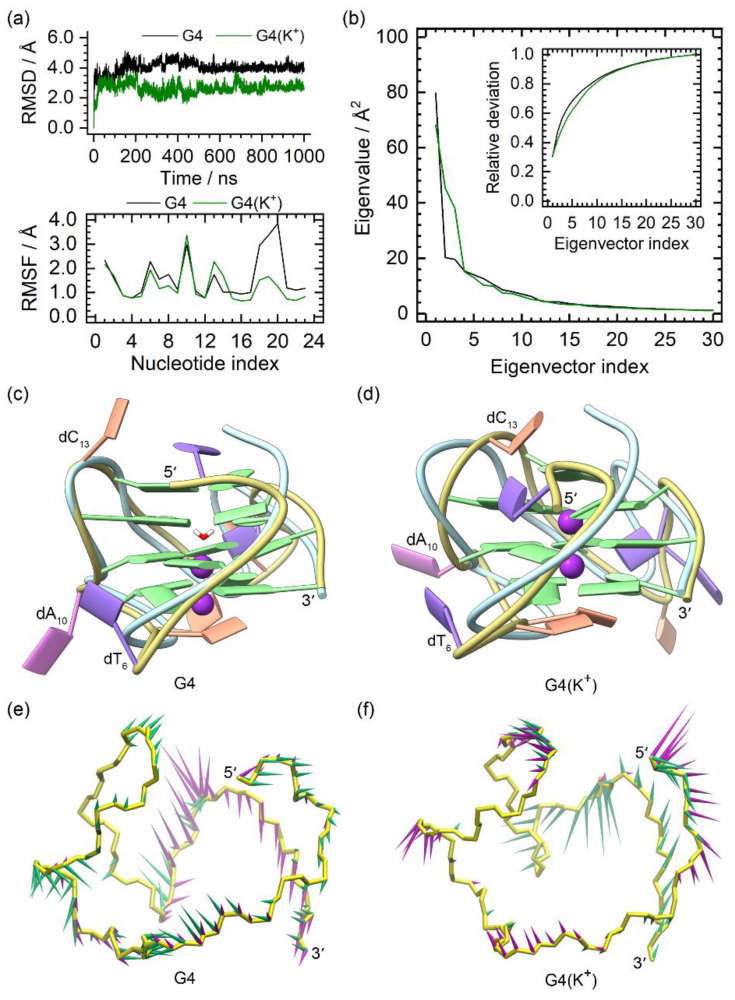
Characteristics of the apo G4 and G4(K^+^). (**a**) root-mean-square deviation (RMSD) and root-mean-square fluctuation (RMSF) profiles of the apo G4 and G4(K^+^); (**b**) eigenvalue profiles constructed by the first 30 eigenvectors of G4 (black) and G4(K^+^) (green) in PCA analysis; (**c**,**d**) superposition of the MD-equilibrated and the NMR structures of *PARP1* G4 and G4(K^+^). The central K^+^ ions in G4(K^+^) are shown as purple spheres. For clarity, the NMR structures are shown as light-blue ribbons; (**e**,**f**) porcupine plots of the dominant motions along the first (green) and the second (magenta) eigenvectors of *PARP1* G4 and G4(K^+^). The direction and size of the colored arrows represent the directions and extents of the principal motions of G4/G4(K^+^) backbone atoms along the given eigenvector.

The MD simulation results of the NMR structures of the *PARP1* G4 and G4(K^+^) are summarized in Figure 2. The smooth RMSD profiles of the G4 and G4(K^+^) indicated their equilibrium states at the late stage (Figure 2a). In agreement with previous reports [29,30], a spontaneous transfer of environmental K^+^ to the central channel of *PARP1* G4 via the bottom passage was observed during the MD simulation, with both sites of the equilibrated G4 occupied by a water molecule and a K^+^ ion, respectively (Figure 2c). For the G4(K^+^), the binding of both central K^+^ ions was stable, as shown in the equilibrated structure (Figure 2d). The structural comparison demonstrated the main conformational variations between the MD-equilibrated structures and the NMR structure of *PARP1* G4 located at the 5′-terminal (dT_1_), the edge-wise loops (dT_6_ and dC_13_), and the double-chain-reversal loop (dC_18_, dT_19_, and dT_20_), in agreement with the RMSF analysis (Figure 2a,c,d). Although dA_10_ was located at the second G-tetrad-forming strand (dG_9_, dG_11_, and dG_12_), it showed exceptionally high RMSF values in both *PARP1* G4 and G4(K^+^) (Figure 2a). This was due to the conformational swing of the dA_10_, which protruded into the environmental solvent instead of packing to the G-tetrads (Figure 2c,d). It should be noted that the fluctuation extent of the double-chain reversal loop in G4(K^+^) was considerably decreased compared to that in the G4 (Figure 2d), indicating the stabilizing effect of the central K^+^ ions. The PCA showed that 40.45% and 42.43% of the essential motions were represented by the first two eigenvectors of the G4 and G4(K^+^), respectively (Figure 2b). Porcupine plots revealed that the first and the second eigenvectors mainly corresponded to the motions of the double-chain-reversal loop in the G4 (Figure 2e). For the G4(K^+^), the first eigenvector mainly represented the motions of the double-chain-reversal loop. By contrast, the second eigenvector mainly represented the motions of the 5′-terminal, dA_10_, and the second edge-wise loop (Figure 2f). The motional features identified were consistent with the RMSF profiles and the conformational comparisons.

### 3.2. Binding Modes of MTR-106 and Talazoparib with PARP1 G4/G4(K^+^)

**Figure 3:**
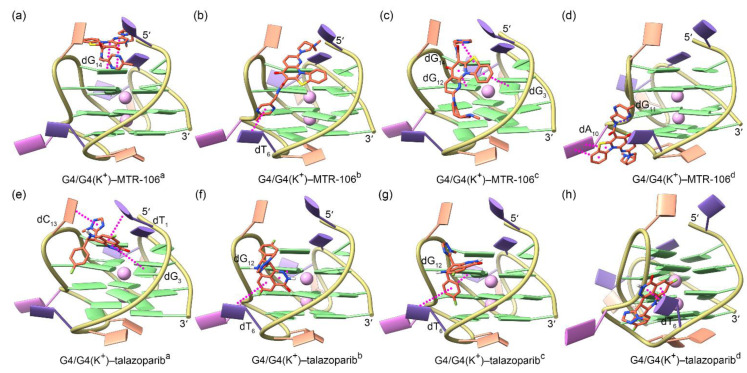
Binding conformations of *PARP1* G4/G4(K^+^) and the ligands of MTR-106 (**a**–**d**) and talazoparib (**e**–**h**) derived from molecular-docking calculations. The central K^+^ ions are shown as faded-pink spheres to indicate their negligible effect on the binding conformations of both ligands. The π–π-stacking and hydrogen-bond interactions between G4/G4(K^+^) and the binding ligands are represented as magenta and blue dotted lines, respectively.

For both the MTR-106 and the talazoparib, the molecular docking identified four potential binding poses that were energetically favored and demonstrated a high probability of binding with *PARP1* G4/G4(K^+^), with the central K^+^ showing a negligible effect on the binding position (Figure 3). For the MTR-106, two binding modes, i.e., end-stacking over the 5′-capping G-pair (Figure 3a) and located at the wide groove (Figure 3b–d), were observed. For the talazoparib, only the groove-binding mode was discovered (Figure 3e–h). As shown in Figure 3 and Table 1, except for the single intermolecular hydrogen bond (Hbond) formed between the amide group of the MTR-106 and the phosphodiester group of the dG_11_ (Figure 3d), both compounds bound to *PARP1* G4/G4(K^+^) via π–π stacking interactions. The binding affinities of the MTR-106 were higher than those of the talazoparib (Table 1), in line with its increased bioactivity. However, no consistent changing trend was found when comparing the binding affinities between the G4–ligand and the G4(K^+^)–ligand (Table 1), which was probably due to the limited accuracy of the docking-energy calculation.

### 3.3. Dynamic Features of the PARP1 G4/G4(K+)–MTR-106/Talazoparib Binding Complexes

As indicated by the RMSDs (Figure S1), all the MD simulations on the *PARP1* G4/G4(K^+^)–MTR-106/talazoparib-binding complexes achieved equilibrium, with the last stage (200 ns at least) presenting no significant oscillations for G4/G4(K^+^) or the binding ligands. In agreement with the apo G4/G4(K^+^), the RMSF profiles of the MTR-106/talazoparib-bound G4/G4(K^+^) showed a similar pattern, i.e., the loop nucleotides presented elevated fluctuations relative to the G-tetrad nucleotides (Figure S2). Except for the pair of G4^a^ and G4(K^+^)^a^, the MTR-106 bound G4(K^+^)s showed reduced fluctuations in the double-chain-reversal loops compared to the corresponding G4s (Figure S2a,b), in line with the stabilizing effect of the central K^+^ found in the apo G4(K^+^). However, the this stabilizing effect was not found in the talazoparib-bound G4/G4(K^+^) structures (Figure S2c,d).

**Figure 4:**
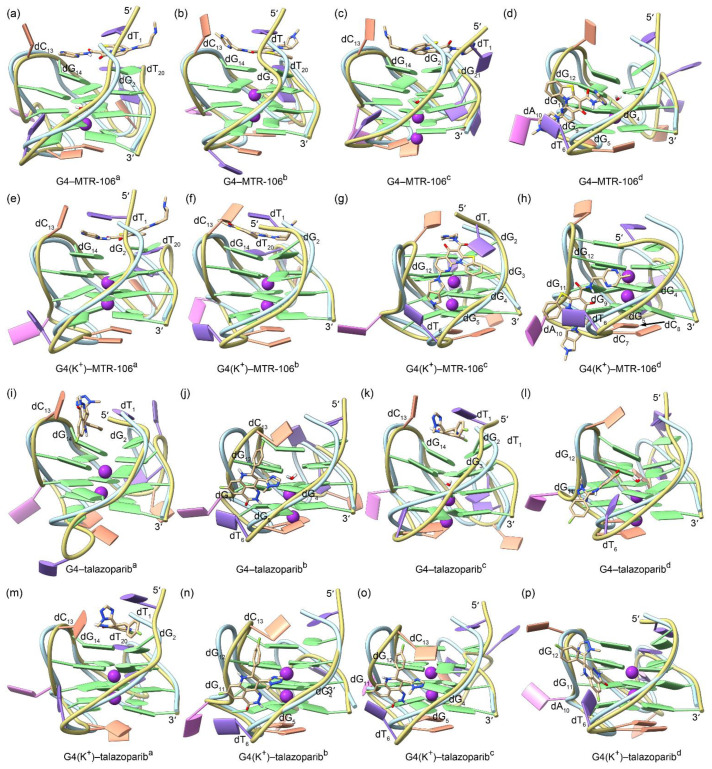
The MD-equilibrated conformations of the *PARP1* G4/G4(K^+^)–MTR-106/talazoparib-binding complexes. For comparison, the structure of NMR *PARP1* G4 is shown as a light-blue ribbon in each panel. The nucleotides that formed close interactions with the binding ligands are labeled.

The equilibrated binding conformations of G4/G4(K^+^)–MTR-106/talazoparib are presented in Figure 4. A comparison with the NMR structure of the *PARP1* G4 revealed that all the MTR-106/talazoparib-bound G4s/G4(K^+^)s maintained the typical feature of a G-quadruplex structure, and the most significant conformational variations occurred at the 5′-terminal and/or the double-chain-reversal loop regions in all the binding complexes, with the exception of G4(K^+^)–MTR-106^b^, in which conformational variations mainly occurred at the first edge-wise loop (Figure 4). The PCA revealed that a minimum of 30.01% of essential motions could be represented by the first two eigenvectors among all the ligand-bound G4/G4(K^+^) structures (Figure S3). The region of the first edge-wise loop in the G4(K^+^)^b^ (Figure S4f) and the regions of the 5′-termini and/or the double-chain-reversal loop in all the other G4s/G4(K^+^)s were demonstrated remarkable motions on the porcupine plots (Figure S4), which was in good accordance with the structural comparisons. Again, environmental potassium ions and water molecules were found to spontaneously enter the central channel of the stabilizer-bound apo G4s during the MD simulations (Figure 4a–d,i–l). For the binding stabilizers, MTR-106^a^ and MTR-106^d^ were located at their initial binding sites in the *PARP1* G4 and G4(K^+^), despite showing some positional changes (Figure 4a,d,e,h). The MTR-106^c^ in the G4(K^+^) also maintained its docking binding conformation (Figure 4g). Notably, the MTR-106^b^ in the G4 and G4(K^+^) and the MTR-106^c^ in the G4 altered their initial groove bindings to stacking over the G-pair, showing some preference for the end-stacking binding mode. Regarding the talazoparib, similar binding-mode alternations were found for the talazoparib^a^ in the G4 and G4(K^+^) and the talazoparib^c^ in the G4, with the other talazoparib in the G4 and G4(K^+^) maintained its original groove bindings (Figure 4i–p). It is worth noting that both the MTR-106 and the talazoparib showed an increased likelihood of altering their original binding mode in binding with the apo G4s, probably due to the greater structural flexibility of the apo G4s relative to the K^+^-stabilized G4(K^+^)s.

**Figure 5:**
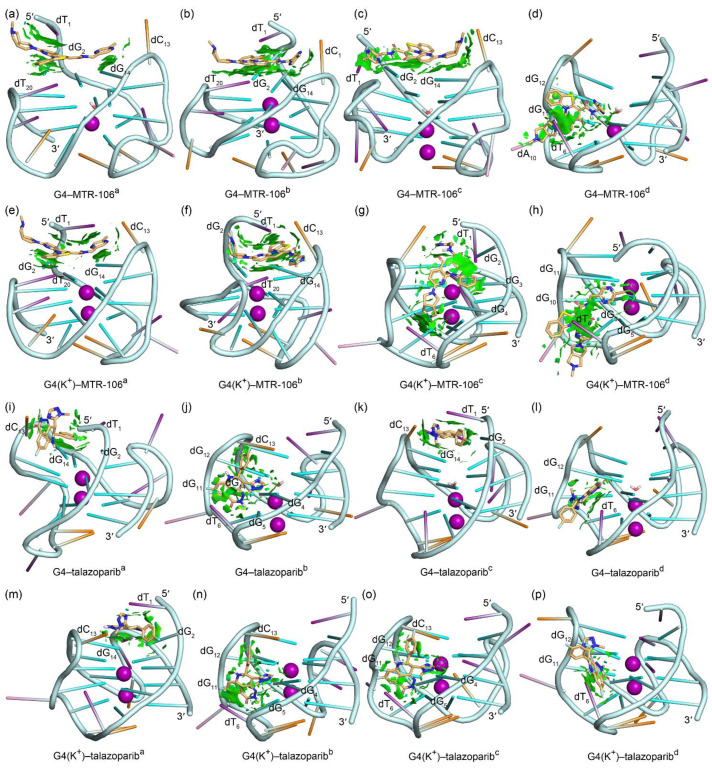
Noncovalent interactions in the MD-equilibrated *PARP1* G4/G4(K^+^)–MTR-106/talazoparib-binding complexes shown as NCI surfaces (isovalue of 0.3 au). The nucleotides involved in the noncovalent interactions are labeled. For clarity, the nucleotide bases of adenine, cytosine, guanine, and thymine are shown as pink, orange, green, and purple sticks, respectively.

To accurately identify the close interactions between the *PARP1* G4 and the binding ligands, a NCIplot analysis that rendered the interactions as isosurfaces was performed; itis shown in Figure 5. The most important binding feature revealed by the NCIplot was the sandwich framework (dT1···MTR-106···G-pair) formed in the end-stacking binding complexes of the G4-MTR-106^a^, G4-MTR-106^b^, G4(K^+^)-MTR-106^a^, and G4(K^+^)-MTR-106^b^ (Figure 5a,b,e,f), which increased the binding affinity of the MTR-106. Although the MTR-106^c^ was stacked over the G-pair in binding with the G4, no sandwich structure was formed (Figure 5c). A common feature in the groove-binding complexes was that all the bound MTR-106s formed vdW interactions with the nucleotide dT_6_ (Figure 5d,g,h). As indicated by the fragmented isosurfaces (Figure 5i–p), the interactions between the talazoparib and the *PARP1* G4/G4(K^+^) were not as extensive as those in the G4/G4(K^+^)-MTR-106. Although the G-pair also played an important role in the end-stacking binding complexes, talazoparib cannot induce the formation of a sandwich framework (Figure 5i,k,m). Furthermore, in addition to the dT_6_ that participated in the interactions with the groove-binding talazoparib, the dG_12_ also made considerable contributions to the binding complexes of G4–talazoparib^b^, G4(K^+^)–talazoparib^b^, and G4(K^+^)–talazoparib^c^ (Figure 5j,l,n–p).

### 3.4. Hydrogen-Bond Analysis of the PARP1 G4/G4(K^+^)–MTR-106/Talazoparib Binding Complexes

**Figure 6:**
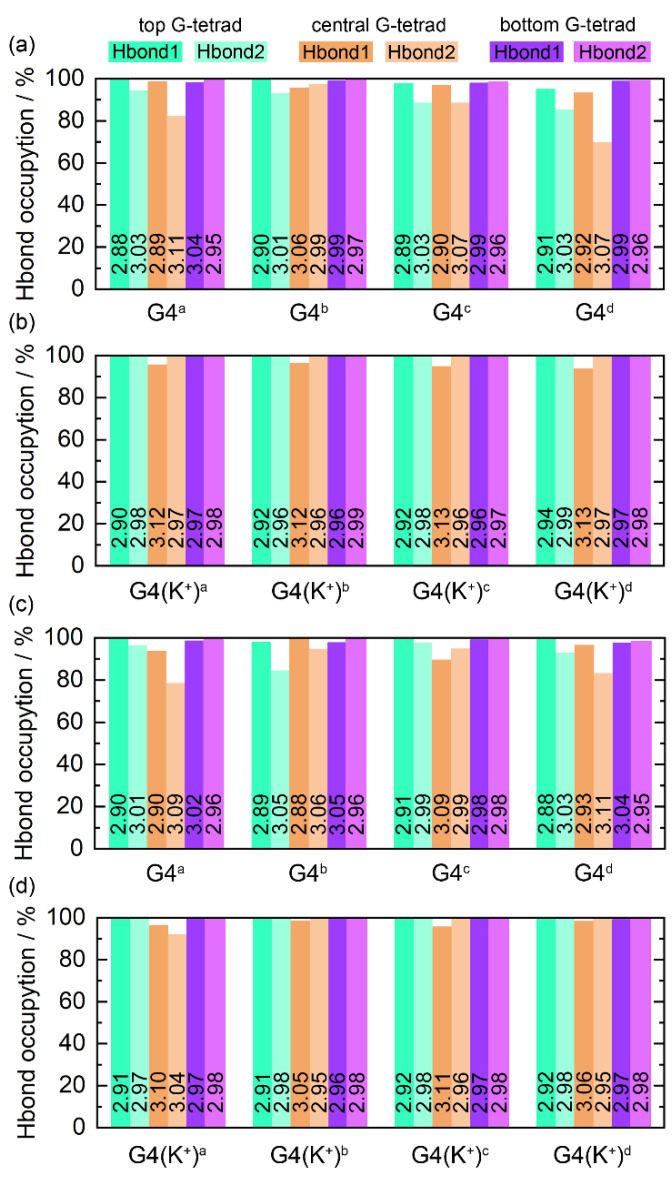
Hoogsteen hydrogen-bond analysis. The hydrogen bonds of N1−H1···O6 and N2−H21···N7 are denoted as Hbond1 and Hbond2, respectively. The number on each column corresponds to the G-tetrad layer-averaged hydrogen-bond length.

The Hoogsteen hydrogen bonds, including N1−H1···O6 (Hbond1) and N2−H21···N7 (Hbond2), formed intramolecularly within the coplanar G-tetrads play an essential role in the maintenance of the structure of G4, the state of which serves as a marker of G4 stability [52]. For each of the ligand-bound *PARP1* G4s, the G-tetrad layer-averaged Hoogsteen hydrogen-bond occupancies (HBOs) and bond lengths are summarized in Figure 6. The HBOs of all G4s/G4(K^+^)s with a minimum of 70.05% signified the structural stability of the *PARP1* G-quadruplex during the MD simulations. A general trend is that all G4(K^+^)s showed increased HBOs compared to their corresponding G4s, indicating the powerful stabilizing effects of the central K^+^ on the G4 structures. Specifically, the minimum HBO of 70.05%, corresponding to the Hbond2 in the central G-tetrad of the MTR-106^d^-bound G4, was increased to 99.61% in the MTR-106^d^-bound G4(K^+^) (Figure 6a,b). The minimum HBO of 78.63%, corresponding to the Hbond2 in the central G-tetrad of the talazoparib^a^-bound G4, was increased to 92.23% in the talazoparib^a^-bound G4(K^+^) (Figure 6c,d).

Intermolecular hydrogen bonds usually make important contributions to biomolecule–ligand interactions. Using the criteria of bond length < 3.5 Å, bond angle > 120°, and occupancy (snapshot percentage of MD trajectory) > 30% [43], a systematic search for intermolecular hydrogen bonds between the *PARP1* G4s/G4(K^+^)s and the binding ligands was performed. As listed in Table 2, the MTR-106^c^ and MTR-106^d^ formed one and two hydrogen bonds with *PARP1* G4(K^+^), respectively. However, an analysis of hydrogen bond length revealed that only the hydrogen bond N2−H22(dG_5_)···O1(MTR-106^d^) was stable (Figure S5a,b). The bond lengths of N2−H22(dG_12_)···N1(MTR-106^c^) were over 3.5 Å during the last 200 ns (Figure S5a), and the bond length of the N2−H22(dG_4_)···N4(MTR-106^d^) showed significant fluctuations during the whole MD simulation (Figure S5b). No stable intermolecular hydrogen bonds were found between the MTR-106 and the *PARP1* G4s. By contrast, more intermolecular hydrogen bonds were found between the *PARP1* G4s/G4(K^+^)s and the binding talazoparib (Table 2). As indicated by the bond-length analysis, the two hydrogen bonds in the G4−talazoparib^d^ binding complexes with low occupancies were unstable (Figure S5e). Nevertheless, all the other intermolecular hydrogen bonds were highly stable (Figure S5c,d,f,g). Notably, except for the hydrogen bond formed between the end-stacked talazoparib^a^ and the *PARP1* G4, all the other intermolecular hydrogen bonds were formed in the groove-binding complexes, indicating that the ligands in the grooves might have had more opportunities or degrees of freedom to alter their binding conformations while the end-stacking ligands gained more binding specificity and stability.

### 3.5. Binding-Free Energies between PARP1 G4 and MTR-106/Talazoparib

To evaluate the binding affinities between the *PARP1* G-quadruplexes and the binding ligands, the binding-free energies were obtained with the MM/GBSA calculation, which has been reported to make good predictions of the hydration-free energy in charged molecules when the relative solvation-free energy is considered [56,57,58]. As shown in Table 3, vdW interaction (∆E_vdW_) was the component that made the greatest contribution to all the binding complexes. A close-to-linear correspondence between the vdW contributions and the binding-free energies (∆G_bind_) was found. By contrast, the contributions from the entropies (T∆S) were the weakest. Furthermore, the net contributions to the overall binding-free energies from the electrostatic interactions (∆E_ele_) and the solvation effects, including the polar part (∆G_GB_) and the non-polar (∆G_SA_) part, were minor.

The MM/GBSA demonstrated that the MTR-106^b^ that formed a sandwiched framework with the dT_1_ and G-pair showed the highest binding strength regardless of whether the central K^+^ initially stabilized the *PARP1* G4 (Table 3). Although the groove-binding MTR-106^d^ (−35.1 kcal/mol) showed a slightly decreased binding affinity with G4(K^+^) relative to the MTR-106^b^ (−36.4 kcal/mol), under the same binding mode, the binding-free energy between the MTR-106^d^ and the G4 was significantly decreased (−21.2 kcal/mol) in comparison with that of the MTR-106^b^ (−35.6 kcal/mol). Therefore, the MTR-106 was the most likely to adopt the end-stacking binding mode via the sandwich framework in binding with *PARP1* G4. When binding with the G4s and G4(K^+^)s, the talazoparib^b^, which was located in the groove, showed the highest binding affinities (−27.1 kcal/mol; Table 3). The structural feature of the alazoparib determined that it had no means with which to form a sandwich framework with *PARP1* G4/G4(K^+^) and, thus, it could not maximize its vdW interactions under the end-stacking binding mode. It is worth noting that both the MTR-106 and the talazoparib showed their highest binding affinities with the *PARP1* G4(K^+^) instead of with the G4, indicating that the central K^+^ ions are beneficial to ligand binding. In addition, the best-performing MTR-106 (−36.4 kcal/mol) demonstrated significant improvements in binding affinity relative to the talazoparib (−27.1 kcal/mol; Table 3), in line with its advanced activity.

**Figure 7:**
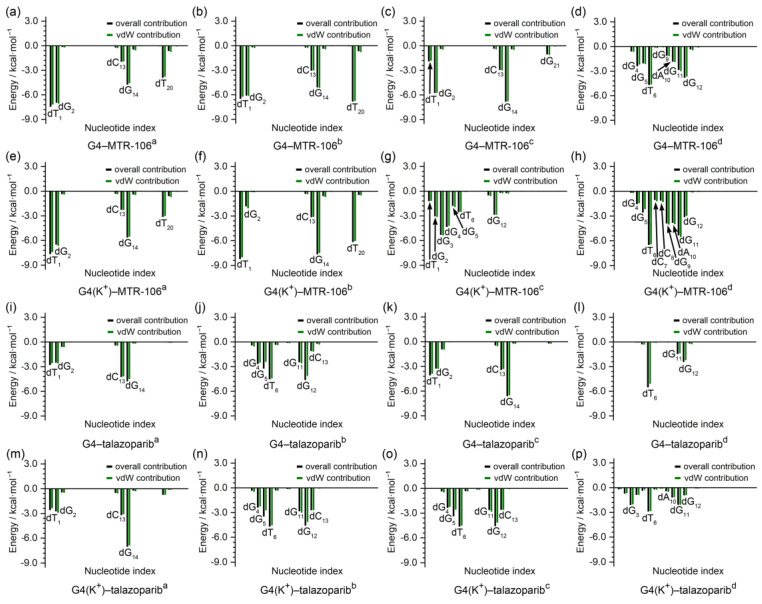
Per-nucleotide decomposition of binding-free energy. The per-nucleotide-based overall and vdW contributions are represented as black and green columns, respectively.

To identify the critical nucleotides of the *PARP1* G-quadruplexes involved in the binding of the MTR-106/talazoparib, a per-residue-decomposition analysis of the binding-free energy was performed. As shown in Figure 7, the G-pair contributed the most in all the end-stacking binding complexes (Figure 7a–c,e,f,i,k,m), and the dT_1_ and dT_20_ also made outstanding contributions in the binding complexes of *PARP1* G4/G4(K^+^)–MTR-106 formed with the sandwich framework (Figure 7a,b,e,f). By contrast, except for the G4(K^+^)–MTR-106^c^, in which a large part of the contributions to the binding originated from the stacking to the dG_3_ and dG_4_ (Figure 5g and Figure 7g), the dT_6_ from the first edge-wise loop generally made the largest contributions in all the groove-binding complexes. It should be noted that the contributions from the vdW interactions were so significant that they were almost equal to the binding-free energies in most cases (Figure 7), further confirming their pivotal role in ligand binding. Additionally, all the key nucleotides identified were in line with the NCIplot-analysis findings (Figure 5 and Figure 7).

### 3.6. Binding Characteristics of PARP1 and MTR-106/Talazoparib

**Figure 8:**
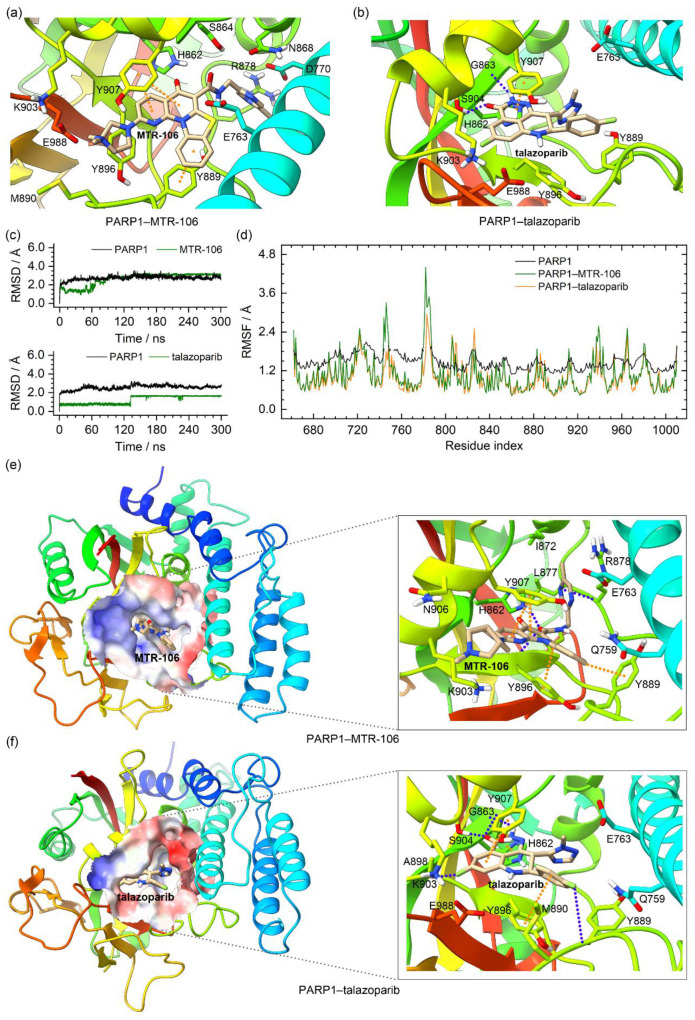
Characteristics of the PARP1–MTR-106/talazoparib-binding complexes. (**a**,**b**) Binding conformations of MTR-106 and talazoparib with PARP1 derived from molecular-docking calculation and the crystal structure, respectively; (**c**) the RMSD profiles of PARP1 and the binding ligands; (**d**) the RMSF profiles of the crystal PARP1 (converted from the B-factor data) and the ligand-bound PARP1; (**e**,**f**) the MD-equilibrated binding conformations of PARP1–MTR-106 and PARP1–talazoparib. The π–π-stacking and hydrogen-bond interactions are represented as orange and blue dotted lines, respectively.

In the crystal structure of the PARP1, the talazoparib formed π–π stacking interactions with Y907 and intermolecular hydrogen bonds with S904 and the backbone of G863. The molecular docking found that the MTR-106 was located at the same site as the talazoparib and mainly formed π–π stacking interactions with Y889 and Y907 (Figure 8a,b). The RMSD analysis of the MD trajectories indicated the binding stability of the MTR-106 and talazoparib with the PARP1 (Figure 8c). The RMSF profiles of the ligand-bound PARP1 showed a similar fluctuation pattern with the crystal structure, validating our MD simulations. The lowered RMSF values may have been a response to the ligands’ stabilizing effect (Figure 8d). Both ligands made some positional adjustments to optimize their interactions with the PARP1 during the MD process. In the equilibrated conformations, the MTR-106 formed π–π stacking interactions with Y889, Y896, and Y907 and intermolecular hydrogen bonds with H862 and the backbones of R878 and Y896 (Figure 8e). The analysis of the occupation and length of the hydrogen bonds indicated that all thre of these hydrogen bonds were generally stable from 150 ns of the MD simulation (Table S1 and Figure S6a). For the talazoparib, π–π stacking interactions with H862, Y896, and Y907 and intermolecular hydrogen bonds with S904 and the backbones of G863 and Y889 were found (Figure 8f). Occupation and bond-length analyses revealed that all the hydrogen bonds except the N−H(Y889)···F2(talazoparib) were stable during the entire MD process (Table S1 and Figure S6b). The PCA calculations revealed that 41.67% and 42.64% of the essential motions were represented by the first two eigenvectors for the MTR-106- and talazoparib-bound PARP1, respectively (Figure S7a), and that the corresponding motions mainly occurred in the peripheral regions of the left and right sides (Figure S7b,c), confirming both ligands’ stabilizing effects on the central region of the PARP1 catalytic domain.

**Figure 9:**
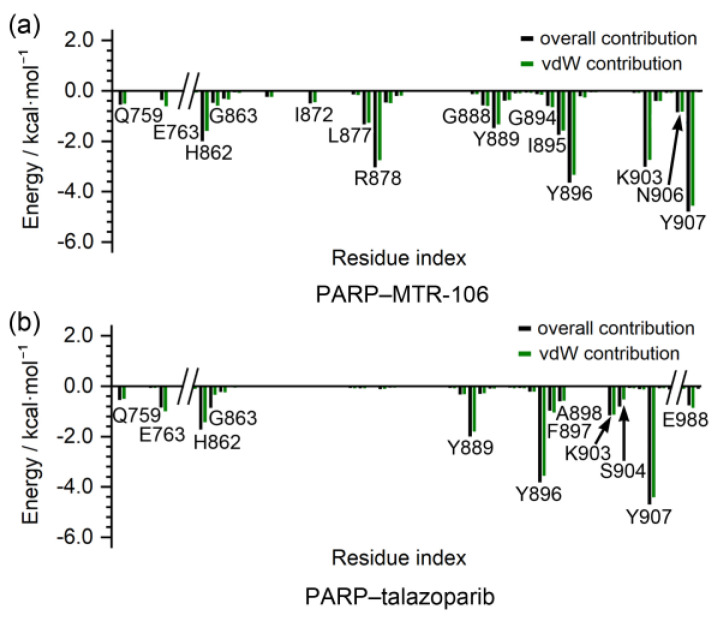
Per-residue decomposition of binding-free energy. The per-residue-based overall and vdW contributions are represented as black and green columns, respectively.

The binding-free energies between the PARP1 and the binding of the MTR-106/talazoparib were calculated with the MM/GBSA method. They were further decomposed to contributing residues to evaluate the binding affinity of the MTR-106 and talazoparib and to identify the critical residues of the PARP1. Similar to the bindings with the *PARP1* G4, the vdW interactions were the components that made the greatest contributions to the bindings with the PARP1, and the MTR-106 (−35.5 kcal/mol) again showed a higher binding affinity than the talazoparib (−26.5 kcal/mol) (Table 4). The free-energy decomposition revealed that the residues involved in the π–π stacking and hydrogen-bond interactions, such as H862, Y889, Y896, and Y907, were important contributors to the binding of both ligands. Notably, L877 and R878, which showed no contribution to the talazoparib binding, contributed significantly to the binding of the MTR-106, and K903 made a significantly increased contribution (Figure 9). All these findings suggest the superior contribution of MTR-106 to inhibitory activity and its effect in a talazoparib-resistant xenograft mouse model.

### 3.7. The ADMET Properties of MTR-106 and Talazoparib

The pharmacokinetic and toxicity profiles of the MTR-106 and talazoparib were predicted by employing the online servers of admetSAR2 and ProTox-II (Table 5). Although they were expected to show different levels of Caco-2 permeability, both compounds were predicted to be orally bioavailable and to be absorbed across the human gastrointestinal lumen into the bloodstream. They may be delivered across the blood–brain barrier and act as P-glycoprotein substrates. The inhibition of the CYP450 enzymes can lead to drug–drug interactions and, thus, increase or reduce therapeutic potency and other pharmacokinetic pathways of co-administered therapeutic agents used in polypharmacy treatment regimes. The MTR-106 showed no inhibitory potency towards CYP450 subtypes and may act as a substrate of CYP3A4. Talazoparib was predicted to show an inhibitory effect against CYP1A2 but lacks the potency to be an inhibitor or substrate of other CYP450 subtypes. The MTR-106 and talazoparib were predicted to have toxic oral doses of 1414 mg/kg and 500 mg/kg, respectively. Both compounds were predicted to be in oral-toxicity class III and IV by admetSAR2 and ProTox-II, respectively, which correspond to largely overlapped LD_50_ ranges (Table 5). The ProTox-II predicted that MTR-106 would show no hepatotoxicity, carcinogenicity, immunotoxicity, or cytotoxicity. However, both the admetSAR2 and the ProTox-II predicted that talazoparib would have a probability of showing hepatotoxicity. Finally, both compounds were predicted to have no effects of eye irritation and corrosion or skin sensitization.

## 4. Discussion

The atomic-level characterization of the binding mode is essential for understanding the interaction mechanism of a drug and its target. In this regard, in silico investigation plays an indispensable role in determining the binding conformation, dynamic feature, and thermodynamic properties, especially when the binding domain in the target macromolecule is unknown [38,59]. AutoDock Vina has been widely applied in molecular-docking calculations; it performs well in reproducing the receptor–ligand binding conformations determined by X-ray crystallography or NMR spectroscopy [60]. However, due to its limited accuracy in evaluating the binding affinity, the most energetically favored conformation is not necessarily the one that fits best with the experimentally determined structure [60]. Therefore, a grid box large enough to encompass *PARP1* G4/G4(K^+^), together with several rounds of docking calculations, were applied to fully sample the binding region of G4/G4(K^+^) and identify the binding poses with energetic preference and high reproducibility. However, the erratic binding affinities of G4/G4(K^+^)–MTR-106/talazoparib contrast with the report that the central K^+^ can increase the binding affinities of G4 ligands [31]. Therefore, MD simulations are desirable for a better assessment of binding conformation and binding-free energy by including the contributions from explicit solvents and induced-fit effects [59].

The long-timescale MD simulations on the *PARP1* G4/G4(K^+^)–MTR-106/talazoparib-binding complexes produced three major findings that are important for understanding *PARP1*-G4-mediated PARP1 inhibition. First, in collaboration with the binding ligand, the central K^+^ can provide an additional stabilizing effect on *PARP1* G4 and enhance the ligand’s binding affinity (Figure 6 and Table 3), which explains the spontaneous transfer of environmental K^+^ to the *PARP1* G4 central channel. This finding agrees with those in a previous report on the roles of central K^+^ in the telomere G4 [31]. Second, a binding-mode alternation from the groove binding to the end-stacking binding was discovered for both the MTR-106 and the talazoparib. However, in contrast to the favored groove binding of talazoparib, MTR-106 prefers stacking with dT1 and the capping G-pair and shows increased binding affinity. Similar observations were reported in the selective binding of anionic phthalocyanine compound and human hybrid (3 + 1) G4s [34], indicating the selective binding mechanism of MTR-106 and *PARP1* G4. Third, as identified by the NCIplot and binding-free-energy decomposition (Figure 5 and Figure 7), vdW interactions are decisive factors for the strength of binding affinity, which is in accordance with the reported G4–ligand-binding systems [33,43]. The stabilizer MTR-106 is a close analog of CX-5461 that shows specific toxicity against BRCA-deficient tumors resistant to PARP inhibition [25]; its high binding strength towards *PARP1* G4 supports its significant antiproliferative activity in HR-deficient and PARPi-resistant cancer cells [27].

In the binding with PARP1, the higher affinity of MTR-106 relative to talazoparib mainly originated from the additional or increased contributions of the residues L877, R878, and K903 via vdW interactions (Figure 8 and Figure 9). In addition, MM/GBSA was evaluated as a reliable method for ranking the relative binding affinities between protein/G4 and binding ligands [58]. The increased binding affinity possibly makes MTR-106 overcome the acquired resistance of talazoparib due to PARP1 mutation and caused it to maintain the trapping state of PARP1 in DNA lesions [19,61].

The pharmacokinetic and toxicity properties of talazoparib have been extensively investigated and well characterized since the FDA approved it in 2018 as a drug for the treatment of BRCA-mutated breast cancer [15]. Similar ADMET profiles were predicted for both compounds. Interestingly, admetSAR2 predicated a better oral bioavailability of MTR-106 (0.5857) relative to talazoparib (0.5429), in line with the bioavailability radar of MTR-106, which showed that all the bioavailability features of the MTR-106 were within the optimum ranges, except for the size (molecular weight of 525.62 g/mol; Figure S8). The predicted ADMET profiles of both compounds agree with their in vivo bioactivities and pharmacokinetic properties [15,27,62].

## 5. Limitations

The major limitation of the current study lies in the research methods of molecular docking and MD simulation. Although additional potential binding poses were sampled in our molecular-docking calculations compared to the standard docking protocol, it is possible that certain binding poses of MTR-106 and talazoparib were missed due to the limited accuracy of AutoDock Vina. As demonstrated by the binding-mode alternation, MD simulations can partly compensate for the defect in molecular docking. This limitation is unlikely to have influenced the conclusion, since the investigation was based on experimentally determined structures, and the findings obtained are in agreement with previously reported experimental results. A further validation of the current findings would need in vitro and/or in vivo investigations on the binding region of *PARP1* G4 for MTR-106 and the direct inhibitory activity of MTR-106 towards PARP1, which will be conducted in our future work.

## 6. Conclusions

Based on the comparative investigation of the binding characteristics of the *PARP1* G-quadruplex and the PARP1 catalytic domain with the ligands of MTR-106 and talazoparib through combined molecular simulations, our in-depth analyses from multiple perspectives revealed that MTR-106 favored end-stacking binding with the *PARP1* G-quadruplex by forming a sandwich framework with the dT_1_ and the capping G-pair, which showed greatly increased binding affinity compared to the talazoparib, which favored the groove-binding mode. Furthermore, in the binding with the PARP1, the MTR-106 formed more extensive interactions with the surrounding PARP1 residues, exhibiting increased binding affinity in comparison with the talazoparib. The high binding ability with *PARP1* G4 and the enhanced inhibition of PARP1 confer advanced bioactivity and attractive antagonistic effects on talazoparib-resistance in PARP1 inhibition upon MTR-106. Overall, the current study provided pivotal insights into the binding and inhibition mechanisms of PARP1, shedding new light on drug design and development targeting the *PARP1* G-quadruplex and PARP1.

## Figures and Tables

**Table 1:** Interactions of MTR-106 and talazoparib with *PARP1* G4 based on molecular docking.

Ligand	Conformation ^1^	Hydrogen Bond	π–π Stacking	Affinity (G4/G4(K^+^)) ^2^
MTR-106	a	-	dG_14_	−7.82/−7.60
	b	-	dT_6_	−7.90/−7.73
	c	-	dG_3_, dG_12_, dG_14_	−8.46/−8.58
	d	dG_11_	dA_10_	−8.01/−7.52
talazoparib	a	-	dT_1_, dG_3_, dC_13_	−6.91/−6.84
	b	-	dT_6_, dG_12_	−6.83/−6.67
	c	-	dT_6_, dG_12_	−6.06/−5.91
	d	-	dT_6_	−6.40/−6.55

^1^ The letters a, b, c, and d indicate the docking conformations of ligands in binding with *PARP1* G4 and G4(K^+^). The conformations are labeled according to their position relative to the capping G-pair of *PARP1* G4; ^2^ affinities between ligand and *PARP1* G4/G4(K^+^) evaluated by AutoDock vina are in kcal∙mol^−1^.

**Table 2:** Intermolecular hydrogen bonds between *PARP1* G4 and the binding ligands.

Model ^1^	Acceptor	Donor	Ocpy ^2^ (%)	Dist ^3^ (Å)	Ang ^4^ (°)
G4(K^+^)–MTR-106 ^c^	MTR-106@N1	dG_12_@H22–N2	57.08	3.16	148.18
G4(K^+^)–MTR-106 ^d^	MTR-106@O1	dG_5_@H22–N2	95.71	2.92	160.90
	MTR-106@N4	dG_4_@H22–N2	49.24	3.16	145.38
G4–talazoparib ^a^	dG_14_@O6	talazoparib@H3–N3	65.24	2.97	159.49
G4–talazoparib ^b^	dG_5_@N3	talazoparib@H1–N1	98.85	2.97	161.86
	talazoparib@O1	dG_5_@H22–N2	96.25	2.99	161.02
	talazoparib@N6	dG_12_@H22–N2	92.44	3.03	150.94
	talazoparib@N2	dG_4_@H22–N2	83.98	3.10	140.54
G4–talazoparib ^d^	talazoparib@O1	dG_12_@H22–N2	32.88	2.98	160.17
	dG_12_@N3	talazoparib@H1–N1	32.69	3.10	153.39
G4(K^+^)–talazoparib ^b^	dG_5_@N3	talazoparib@H1–N1	98.24	2.98	161.26
	talazoparib@O1	dG_5_@ H22–N2	93.30	3.02	158.85
	talazoparib@N6	dG_12_@ H22–N2	88.54	3.04	149.11
	talazoparib@N2	dG_4_@H22–N2	65.31	3.04	135.88
G4(K^+^)–talazoparib ^c^	dG_5_@N3	talazoparib@H1–N1	95.68	2.97	161.89
	talazoparib@O1	dG_5_@H22–N2	90.86	3.02	160.43
	talazoparib@N6	dG_12_@H22–N2	88.03	3.04	147.75
	talazoparib@N2	dG_4_@H22–N2	60.19	3.04	133.66
	alazoparib@N6	dG_4_@H22–N2	42.51	3.23	139.98

^1^ The superscripted letters a, b, c, and d indicate the initial conformations of ligands in binding with *PARP1* G4 and G4(K^+^); ^2^ Ocpy means hydrogen bond occupancy; ^3^ Dist means hydrogen bond distance; ^4^ Ang means hydrogen-bond angle.

**Table 3:** Binding-free energies between *PARP1* G4/G4(K^+^) and the binding ligands.

Receptor	Ligand ^1^	Energy Components ^2^						
		ΔE_ele_	ΔE_vdW_	ΔG_GB_	ΔG_SA_	ΔH	TΔS	ΔG_bind_
G4	MTR-106 ^a^	2.4 ± 1.5	−54.1 ± 3.0	1.9 ± 1.3	−6.0 ± 0.2	−55.8 ± 3.0	21.8 ± 9.2	−34.0
	MTR-106 ^b^	4.6 ± 1.3	−59.6 ± 2.5	3.1 ± 1.1	−5.8 ± 0.2	−57.7 ± 2.4	22.1 ± 10.1	−35.6
	MTR-106 ^c^	0.3 ± 3.6	−41.0 ± 7.8	3.1 ± 3.0	−4.5 ± 0.7	−42.0 ± 8.3	22.2 ± 10.4	−19.8
	MTR-106 ^d^	−2.7 ± 2.3	−41.2 ± 9.3	5.5 ± 4.1	−4.3 ± 3.0	−42.7 ± 7.4	21.5 ± 10.2	−21.2
G4(K^+^)	MTR-106 ^a^	0.8 ± 1.7	−55.2 ± 3.7	3.8 ± 1.6	−6.2 ± 0.4	−56.8 ± 3.8	22.1 ± 10.4	−34.7
	MTR-106 ^b^	−3.1 ± 1.4	−58.0 ± 3.1	6.8 ± 1.3	−5.9 ± 0.3	−60.2 ± 3.0	23.8 ± 10.4	−36.4
	MTR-106 ^c^	−4.7 ± 1.1	−46.0 ± 3.5	8.2 ± 1.0	−5.0 ± 0.3	−47.6 ± 3.6	25.1 ± 11.1	−22.5
	MTR-106 ^d^	−3.3 ± 1.2	−60.4 ± 3.7	7.8 ± 1.0	−6.6 ± 0.3	−62.6 ± 3.6	27.5 ± 10.5	−35.1
G4	talazoparib ^a^	−7.2 ± 1.3	−31.5 ± 3.9	9.2 ± 1.1	−4.0 ± 0.4	−33.5 ± 4.0	18.0 ± 9.3	−15.5
	talazoparib ^b^	−1.7 ± 1.3	−38.4 ± 3.1	3.6 ± 1.1	−4.7 ± 0.3	−41.2 ± 2.9	17.2 ± 9.4	−24.0
	talazoparib ^c^	−5.4 ± 1.6	−39.0 ± 3.2	8.2 ± 1.3	−4.5 ± 0.3	−40.8 ± 3.2	17.8 ± 8.7	−23.0
	talazoparib ^d^	−2.0 ± 1.8	−20.0 ± 2.6	3.3 ± 1.5	−2.7 ± 0.2	−21.2 ± 2.3	16.9 ± 8.9	−4.3
G4(K^+^)	talazoparib ^a^	−3.9 ± 1.4	−36.0 ± 3.5	6.5 ± 1.1	−4.1 ± 0.3	−37.5 ± 2.8	18.0 ± 8.7	−19.5
	talazoparib ^b^	−2.1 ± 0.9	−42.0 ± 3.0	4.2 ± 0.8	−4.8 ± 0.2	−44.7 ± 3.0	17.6 ± 9.5	−27.1
	talazoparib ^c^	−2.2 ± 1.0	−41.0 ± 2.9	4.2 ± 0.8	−4.8 ± 0.2	−43.7 ± 2.8	18.5 ± 9.2	−25.2
	talazoparib ^d^	−2.3 ± 2.6	−25.1 ± 8.3	4.5 ± 3.0	−3.2 ± 1.1	−26.1 ± 8.7	16.6 ± 9.1	−9.5

^1^ The superscripted letters a, b, c, and d indicate the initial conformations of ligands in binding with *PARP1* G4 and G4(K^+^); ^2^ energies are in kcal∙mol^−1^.

**Table 4:** Binding-free energies between PARP1 and the binding ligands.

Receptor	Ligand	Energy Components ^1^						
		ΔE_ele_	ΔE_vdW_	ΔG_GB_	ΔG_SA_	ΔH	TΔS	ΔG_bind_
PARP1	MTR-106	−4.0 ± 1.1	−58.4 ± 2.7	8.3 ± 0.9	−7.5 ± 0.2	−61.6 ± 2.7	26.1 ± 13.0	−35.5
PARP1	talazoparib	−7.0 ± 1.0	−43.3 ± 2.8	8.7 ± 0.7	−5.6 ± 0.1	−47.2 ± 2.6	20.7 ± 11.4	−26.5

^1^ Energies are in kcal∙mol^−1^.

**Table 5:** The ADMET properties of MTR-106 and talazoparib.

Property	MTR-106		Talazoparib	
	AdmetSAR2	ProTox-II	AdmetSAR2	ProTox-II
Human oral bioavailability	0.5857	-	0.5429	-
GI ^1^ absorption	Yes (0.9922)	-	Yes (0.9951)	-
Caco-2 permeability ^2^	No (0.7863)	-	Yes (0.6579)	-
BBB ^3^	Yes (0.9000)	-	Yes (0.8000)	-
P-gp ^4^ substrate	Yes (0.8047)	-	Yes (0.5664)	-
CYP1A2 ^5^ inhibitor	No (0.5952)	-	Yes (0.8200)	-
CYP2C9 ^5^ inhibitor	No (0.6741)	-	No (0.6486)	-
CYP2C19 ^5^ inhibitor	No (0.5853)	-	No (0.5888)	-
CYP2D6 ^5^ inhibitor	No (0.8830)	-	No (0.9394)	-
CYP3A4 ^5^ inhibitor	No (0.6878)	-	No (0.6895)	-
CYP2C9 ^5^ substrate	No (1.0000)	-	No (0.8038)	-
CYP2D6 ^5^ substrate	No (0.8202)	-	No (0.8402)	-
CYP3A4 ^5^ substrate	Yes (0.6977)	-	No (0.6895)	-
Oral LD_50_ ^6^	-	1414	-	500
Oral-toxicity class	III ^7^	IV ^8^	III ^7^	IV ^8^
Acute oral toxicity ^9^	2.008	-	2.632	-
Hepatotoxicity	Yes (0.7176)	No (0.60)	Yes (0.5803)	Yes (0.63)
Carcinogenicity	No (0.8600)	No (0.58)	No (0.8857)	No (0.54)
Immunotoxicity	-	No (0.92)	-	No (0.73)
Mutagenicity	-	Yes (0.54)	-	No (0.57)
Cytotoxicity	-	No (0.60)	-	No (0.81)
Eye irritation and corrosion	No	-	No	-
Skin sensitization	No (0.8543)	-	No (0.9017)	-

^1^ GI means gastrointestinal, numbers in parentheses of this table represent the prediction probability; ^2^ Caco-2 means human colorectal-adenocarcinoma cell; ^3^ BBB means blood–brain barrier; ^4^ P-gp means P-glycoprotein; ^5^ represents various subtypes of the CYP450 metabolizing enzyme; ^6^ LD_50_ represents the dose required to kill 50% of the test subjects, the unit is mg/kg; ^7^ Oral-toxicity class III is equivalent to LD_50_ range of 500 mg/kg < LD_50_ ≤ 5000 mg/kg; ^8^ Oral-toxicity class IV is equivalent to LD50 range of 300 mg/kg < LD_50_ ≤ 2000 mg/kg; ^9^ the unit is log(1/(mol∙kg^−1^)).

## Data Availability

The data are contained within this article.

## Supplementary Materials

The following supporting information can be downloaded at: https://www.mdpi.com/article/10.3390/molecules28041829/s1, Table S1. Intermolecular hydrogen bonds between PARP1 and the binding ligands. Figure S1. RMSD profiles of the *PARP1* G4/G4(K^+^)–MTR-106/talazoparib binding complexes. The subscripted words a, b, c, and d indicate the initial conformations of ligands in their binding with *PARP1* G4s and G4(K^+^)s. Figure S2. RMSF profiles of the MTR-106/talazoparib bound *PARP1* G4s/G4(K^+^)s. The subscripted words a, b, c, and d indicate the *PARP1* G4s and G4(K^+^)s with the binding ligands showing corresponding initial conformations. Figure S3. Eigenvalue profiles constructed by the first 30 eigenvectors of the ligand bound G4s/G4(K^+^)s in PCA analysis. The subscripted words a, b, c, and d indicate the initial conformations of ligands in their binding with *PARP1* G4s and G4(K^+^)s, and the eigenvalue profiles of the corresponding G4s/G4(K^+^)s are colored in black, green, orange, and purple, respectively. Figure S4. Porcupine plots of the dominant motions along the first (green) and the second (magenta) eigenvectors of *PARP1* G4s and G4(K^+^)s. The direction and size of the colored arrows represent the directions and extents of the principal motions of G4/G4(K^+^) backbone atoms along the certain eigenvector. The subscripted words a, b, c, and d indicate the initial conformations of ligands in their binding with *PARP1* G4s and G4(K^+^)s. Figure S5. Hydrogen bond length fluctuation along the MD simulations. The subscripted words a, b, c, and d indicate the initial conformations of ligands in their binding with *PARP1* G4s and G4(K^+^)s. Figure S6. Hydrogen bond length fluctuation along the MD simulations. Figure S7. PCA result of the PARP1–MTR-106/talazoparib binding complexes. (a) eigenvalue profiles constructed by the first 30 eigenvectors of the ligand bound PARP1. (b) and (c) porcupine plots of the dominant motions along the first (green) and the second (magenta) eigenvectors of the MTR-106 and talazoparib bound PARP1, respectively. The direction and size of the colored arrows represent the directions and extents of the principal motions of PARP1 backbone atoms along the certain eigenvector. Figure S8. The bioavailability radar of MTR-106 and talazoparib. The colored zone indicates the appropriate physicochemical region for oral bioavailability. The optimum ranges for bioavailability for each feature are: Lipophilicity (LIPO), XLOGP3 ranges from 0.7 to 5.0; Size (SIZE), molecular weight ranges from 150 g/mol to 500 g/mol; Polarity (POLAR), TPSA ranges from 20 Å^2^ to 130 Å^2^; Insolubility (INSOLU), logS(ESOL) should be less than 6.0; INSATU, fraction of carbon atoms in sp3 hybridization should be more than 0.25; Flexibility (FLEX), number of rotatable bonds should be less than 9.
