# Supplementary material for: In Silico Investigation of the Molecular Mechanism of PARP1 Inhibition for the Treatment of BRCA-Deficient Cancers
Source: Molecules. 2023 Feb 15;28(4):1829. doi: 10.3390/molecules28041829 (PMC9961911; doi:10.3390/molecules28041829)
Supplement: Supplementary file 1 [file molecules-28-01829-s001.zip › molecules-2180862-supplementary.pdf]

In Silico Investigation of the Molecular Mechanism of PARP1 Inhibition for the Treatment of BRCA-Deficient Cancers

Fengqin Yan[1], Zhenfu Fu[1], Guo Li[2,3,*], Zhiguo Wang[4,*]

[1] Department of Radiotherapy, The Cancer Hospital of the University of Chinese Academy of Sciences (Zhejiang Cancer Hospital), Institute of Basic Medicine and Cancer (IBMC), Chinese Academy of Sciences, Hangzhou, Zhejiang Province, 310022, China; yanfq@zjcc.org.cn (F.Y.); fuzf1000@163.com (Z.F.)

[2] Department of Biochemistry and Molecular Biology, Hainan Medical University, Haikou, Hainan Province, 571199, China; liguo2000@aliyun.com

[3] Hainan Province Clinical Medical Center, Hainan Hospital Affiliated to Hainan Medical University, Haikou, Hainan Province, 571199, China; liguo2000@aliyun.com

[4] Institute of Ageing Research, School of Basic Medical Sciences, Hangzhou Normal University, Hangzhou 311121, China; zhgwang@aliyun.com

Tables

Table S1. Intermolecular hydrogen bonds between PARP1 and the binding ligands.

Model	Acceptor	Donor	Ocpy[1] (%)	Dist[2] (Å)	Ang[3] (°)
PARP1–MTR-106	MTR-106@O2	Y_{896}@ H–N	64.04	3.10	164.13
	MTR-106@O2	H_{867}@ HE2–NE2	37.68	3.11	159.27
	MTR-106@N5	R_{878}@H–N	32.91	3.29	160.88
PARP1–talazoparib	talazoparib@O1	S_{904}@HG–OG	94.61	2.81	161.70
	G_{863}@O	talazoparib@H1–N1	98.53	2.97	159.10
	talazoparib@O1	G_{863}@H–N	90.86	2.91	151.02
	talazoparib@F2	Y_{889}@H–N	27.74	3.21	155.05

[1] Ocpy means hydrogen bond occupancy.

[2] Dist means hydrogen bond distance.

[3] Ang means hydrogen bond angle.

Figures

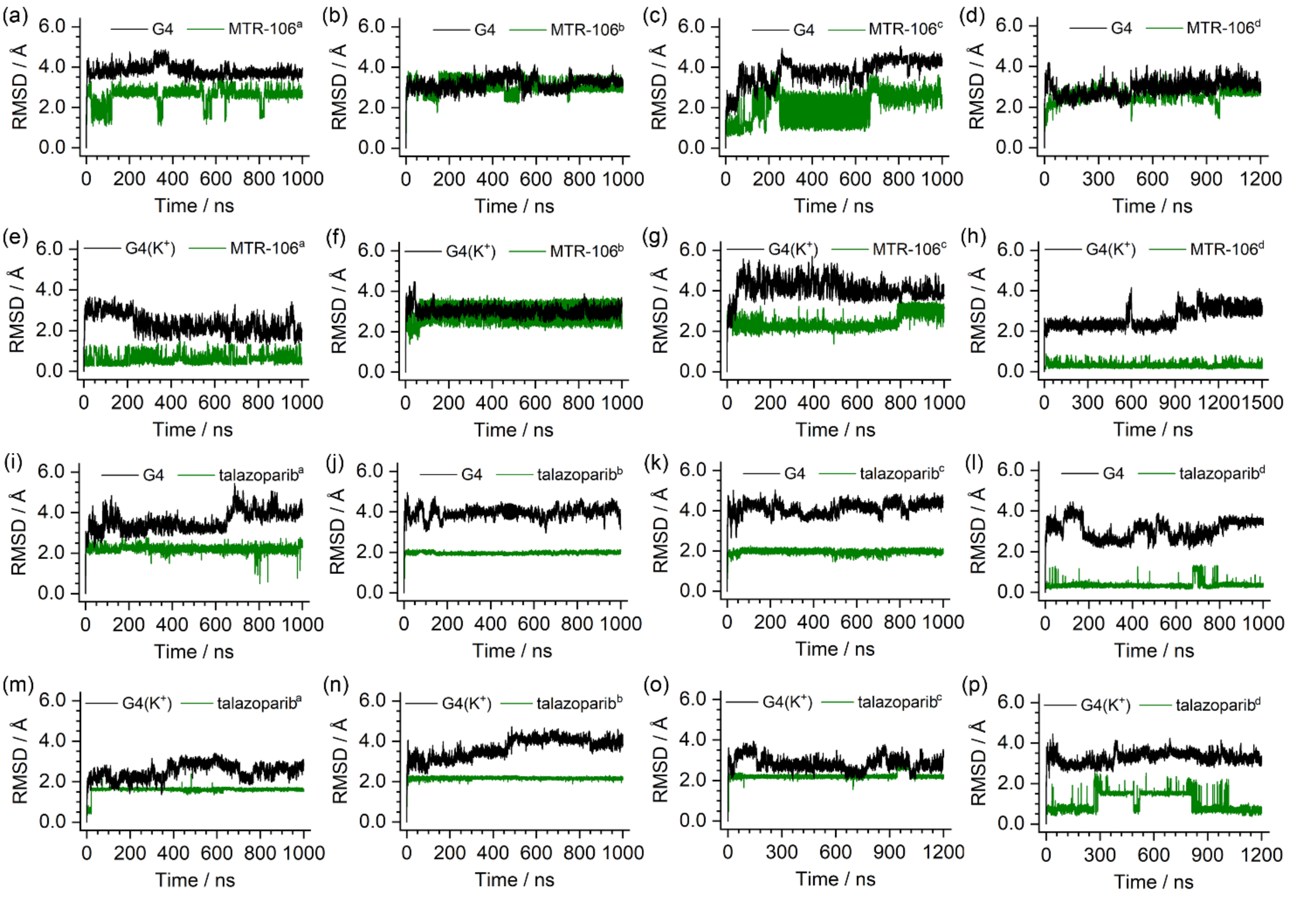

Figure S1. RMSD profiles of the *PARP1* G4/G4(K^+)–MTR-106/talazoparib binding complexes. The subscripted words a, b, c, and d indicate the initial conformations of ligands in their binding with *PARP1* G4s and G4(K^+)s.

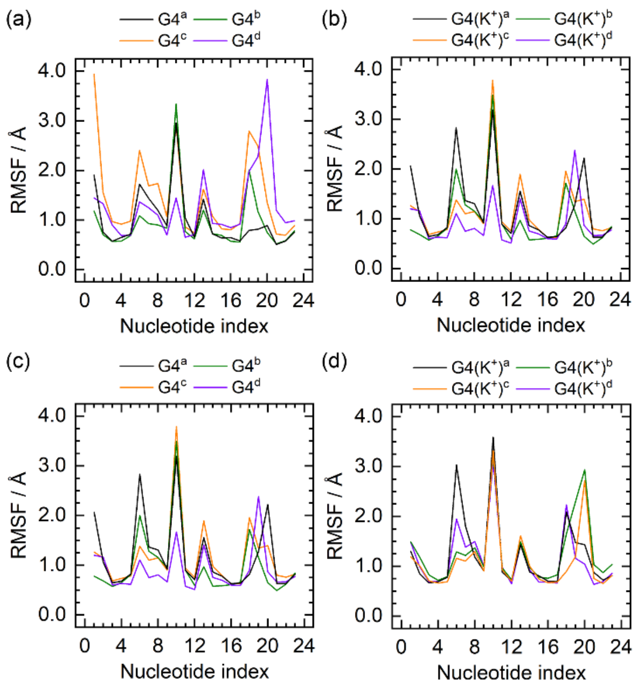

Figure S2. RMSF profiles of the MTR-106/talazoparib bound *PARP1* G4s/G4(K^+)s. The subscripted words a, b, c, and d indicate the *PARP1* G4s and G4(K^+)s with the binding ligands showing corresponding initial conformations.

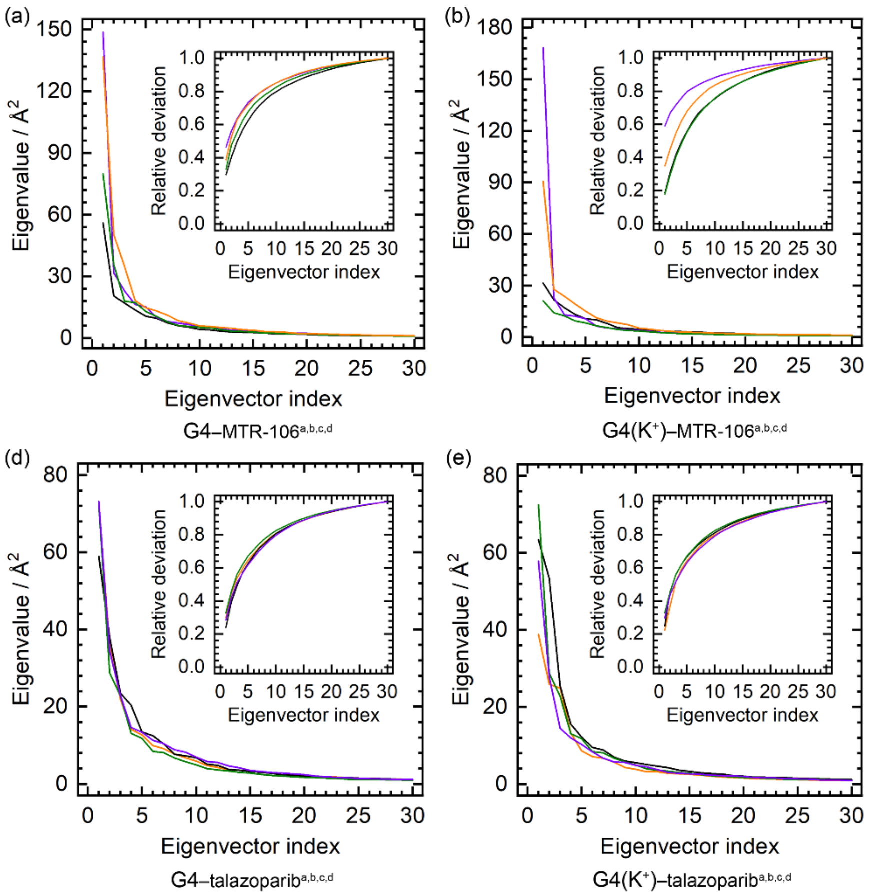

Figure S3. Eigenvalue profiles constructed by the first 30 eigenvectors of the ligand bound G4s/G4(K^+)s in PCA analysis. The subscripted words a, b, c, and d indicate the initial conformations of ligands in their binding with *PARP1* G4s and G4(K^+)s, and the eigenvalue profiles of the corresponding G4s/G4(K^+)s are colored in black, green, orange, and purple, respectively.

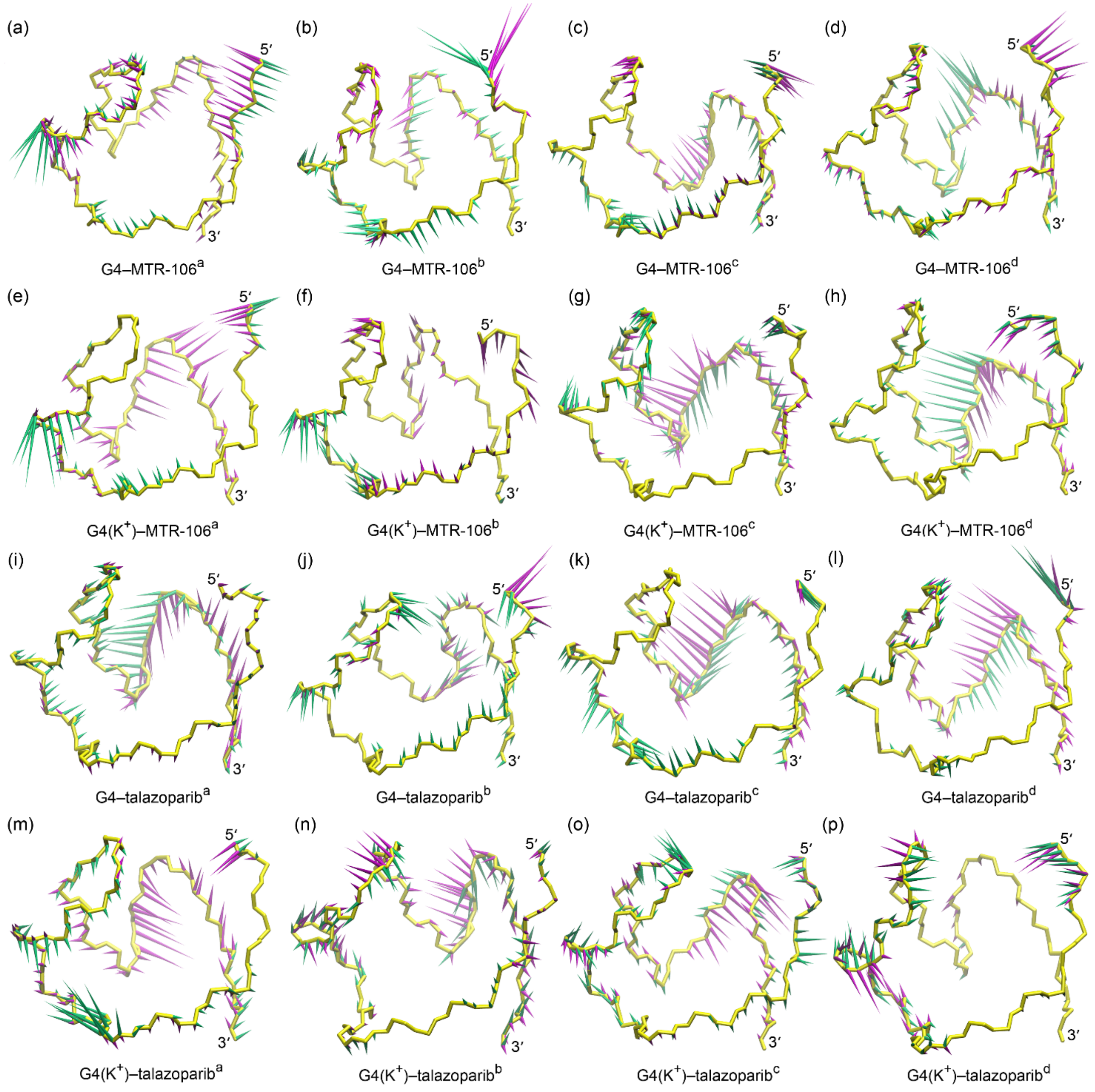

Figure S4. Porcupine plots of the dominant motions along the first (green) and the second (magenta) eigenvectors of *PARP1* G4s and G4(K^+)s. The direction and size of the colored arrows represent the directions and extents of the principal motions of G4/G4(K^+) backbone atoms along the certain eigenvector. The subscripted words a, b, c, and d indicate the initial conformations of ligands in their binding with *PARP1* G4s and G4(K^+)s.

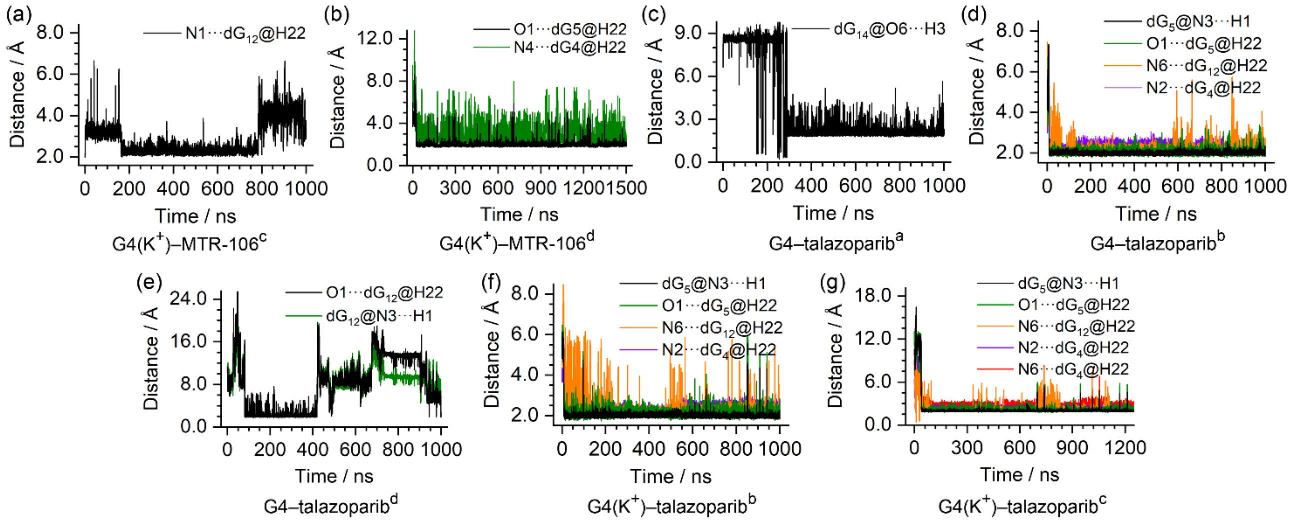

Figure S5. Hydrogen bond length fluctuation along the MD simulations. The subscripted words a, b, c, and d indicate the initial conformations of ligands in their binding with *PARP1* G4s and G4(K^+)s.

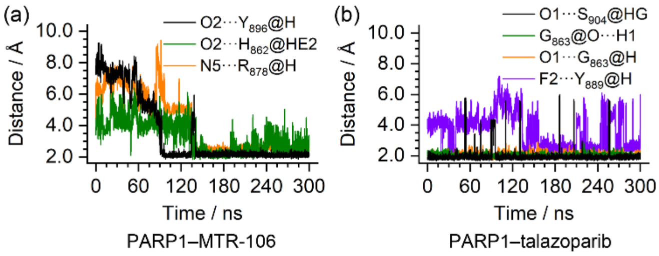

Figure S6. Hydrogen bond length fluctuation along the MD simulations.

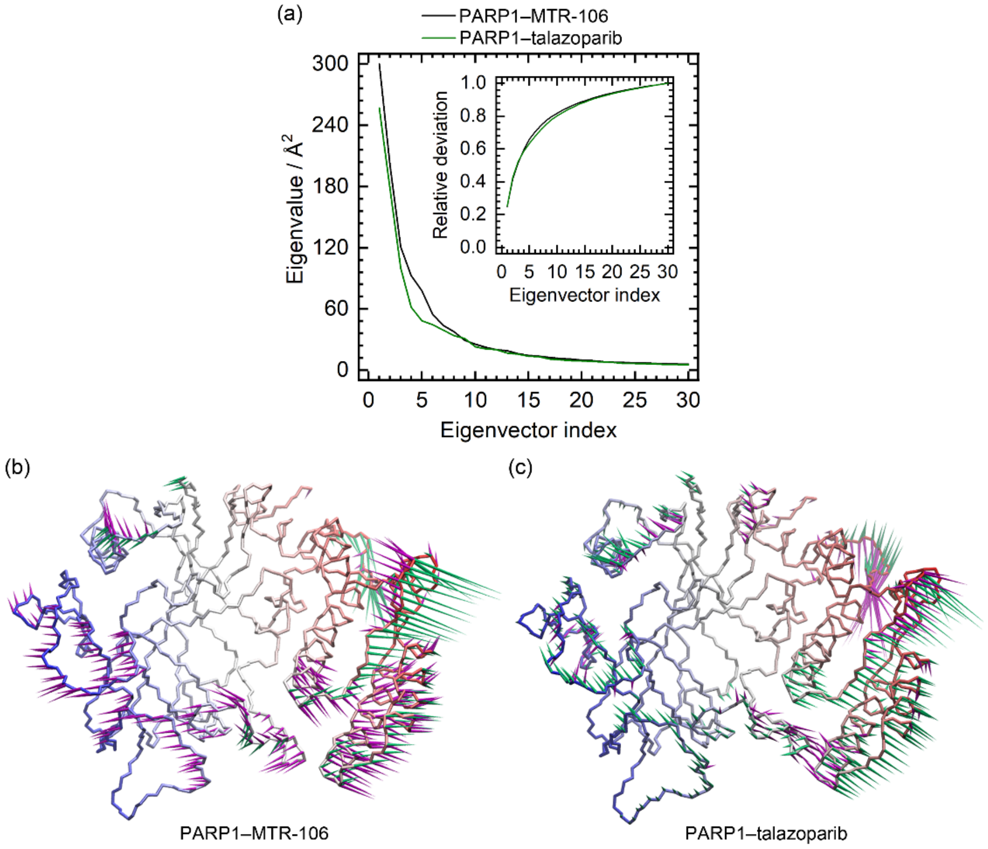

Figure S7. PCA result of the PARP1–MTR-106/talazoparib binding complexes. (a) eigenvalue profiles constructed by the first 30 eigenvectors of the ligand bound PARP1. (b) and (c) porcupine plots of the dominant motions along the first (green) and the second (magenta) eigenvectors of the MTR-106 and talazoparib bound PARP1, respectively. The direction and size of the colored arrows represent the directions and extents of the principal motions of PARP1 backbone atoms along the certain eigenvector.

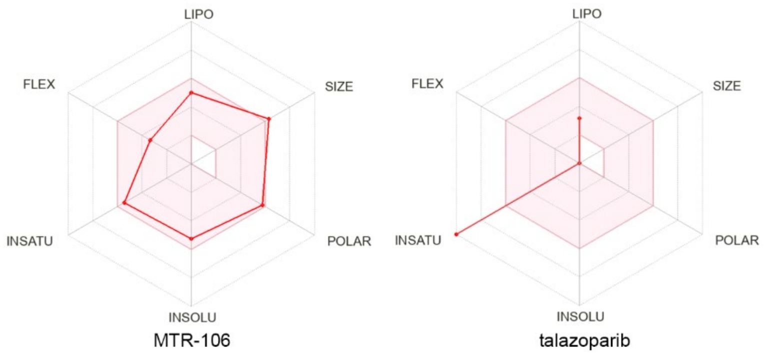

Figure S8. The bioavailability radar of MTR-106 and talazoparib. The colored zone indicates the appropriate physicochemical region for oral bioavailability. The optimum ranges for bioavailability for each feature are: Lipophilicity (LIPO), XLOGP3 ranges from 0.7 to 5.0; Size (SIZE), molecular weight ranges from 150 g/mol to 500 g/mol; Polarity (POLAR), TPSA ranges from 20 Å^2 to 130 Å^2; Insolubility (INSOLU), logS(ESOL) should be less than 6.0; INSATU, fraction of carbon atoms in sp3 hybridization should be more than 0.25; Flexibility (FLEX), number of rotatable bonds should be less than 9.
